# Optimization of a direct Serum Response Factor Inhibitor Peptide

**DOI:** 10.51894/001c.143895

**Published:** 2025-10-30

**Authors:** Melissa Meschkewitz, Erika M. Lisabeth, Richard R. Neubig

**Affiliations:** 1 College of Osteopathic Medicine Michigan State University

## 12

Approximately 18.6 million deaths worldwide were attributed to cardiovascular disease (CVD) in 2019; in the US alone the estimated direct and indirect cost of CVD for 2016 to 2017 was $363 billion. Cardiac fibrosis, a condition leading to increased cardiac stiffness and decreased cardiac plasticity, is one of the main contributing factors of CVD. The hallmark of cardiac fibrosis is activation of cardiac fibroblasts to myofibroblasts and changes in the extracellular matrix resulting in increased tissue stiffness. Serum Response Factor (SRF) and its co-activator myocardin-related transcription factor A (MRTF-A) is one of the key pathways regulating myofibroblast activation. Increased MRTF-A/SRF signaling has been shown to active cardiac fibroblast to myofibroblasts. Despite the involvement of SRF over-activation through MRTFA/SRF signaling in multiple disease pathways, no direct inhibitor of SRF has been identified. A small 21-mer peptide, the B1 box motif of MRTF-A, can bind to SRF and effectively inhibit MRTF-A/SRF complex formation in vitro. In this research, we aim to optimize the binding affinity of the 21-mer peptide to SRF and its ability to inhibit MRTF-A/SRF complex formation through computational and biochemical techniques. We utilized Amplified Luminescence Homogenous Proximity Assay (ALPHA) to develop a high throughput assay to screen different peptides against the MRTF-A/SRF complex. We determined that a 14-mer peptide has almost an identical IC50 compared to the 21-mer peptide in our assay, 1.6µM and 1.3µM, respectively. This suggests that a shorter, and perhaps more drug-like peptide may be effective in disrupting this protein:protein interaction. Additionally, we explored different mutations identified through previous research or computational analysis that indicated that a single tyrosine to phenylalanine mutation can increase the potency in our biochemical assay almost 2-fold, resulting in an IC50 of 0.5µM for the 21-mer peptide with phenylalanine substitution.

This work was supported by the AHA predoctoral fellowship 23PRE1019204.

